# Regenerative Cardiovascular Therapies: Stem Cells and Beyond

**DOI:** 10.3390/ijms20061420

**Published:** 2019-03-21

**Authors:** Bernhard Wernly, Moritz Mirna, Richard Rezar, Christine Prodinger, Christian Jung, Bruno K. Podesser, Attila Kiss, Uta C. Hoppe, Michael Lichtenauer

**Affiliations:** 1 Clinic of Internal Medicine II, Department of Cardiology, Paracelsus Medical University of Salzburg, 5020 Salzburg, Austria; m.mirna@salk.at (M.M.); r.rezar@salk.at (R.R.); u.hoppe@salk.at (U.C.H.); m.lichtenauer@salk.at (M.L.); 2 Department of Dermatology, Paracelsus Medical University of Salzburg, 5020 Salzburg, Austria; c.prodinger@salk.at; 3 Division of Cardiology, Pulmonology and Vascular Medicine, Medical Faculty, University of Düsseldorf, 40225 Düsseldorf, Germany; Christian.Jung@med.uni-duesseldorf.de; 4 Ludwig Boltzmann Cluster for Cardiovascular Research, Center for Biomedical Research, Medical University Vienna, 1090 Vienna, Austria; bruno.podesser@meduniwien.ac.at (B.K.P.); attila.kiss@meduniwien.ac.at (A.K.)

**Keywords:** regenerative cardiovascular therapy, stem cell, myocardial infarction, miRNA, heart failure, reperfusion injury, conditioning

## Abstract

Although reperfusion therapy has improved outcomes, acute myocardial infarction (AMI) is still associated with both significant mortality and morbidity. Once irreversible myocardial cell death due to ischemia and reperfusion sets in, scarring leads to reduction in left ventricular function and subsequent heart failure. Regenerative cardiovascular medicine experienced a boost in the early 2000s when regenerative effects of bone marrow stem cells in a murine model of AMI were described. Translation from an animal model to stem cell application in a clinical setting was rapid and the first large trials in humans suffering from AMI were conducted. However, high initial hopes were early shattered by inconsistent results of randomized clinical trials in patients suffering from AMI treated with stem cells. Hence, we provide an overview of both basic science and clinical trials carried out in regenerative cardiovascular therapies. Possible pitfalls in specific cell processing techniques and trial design are discussed as these factors influence both basic science and clinical outcomes. We address possible solutions. Alternative mechanisms and explanations for effects seen in both basic science and some clinical trials are discussed here, with special emphasis on paracrine mechanisms via growth factors, exosomes, and microRNAs. Based on these findings, we propose an outlook in which stem cell therapy, or therapeutic effects associated with stem cell therapy, such as paracrine mechanisms, might play an important role in the future. Optimizing stem cell processing and a better understanding of paracrine signaling as well as its effect on cardioprotection and remodeling after AMI might improve not only AMI research, but also our patients’ outcomes.

## 1. Introduction

At the end of the 19th century, correlations between thrombotic occlusion of coronary arteries and the presence of myocardial infarction were postulated [1]. Almost at the same time, the Dutch scientist and later Nobel laureate Willem Einthoven developed the electrocardiogram, which today is indispensable in clinical routine. As early as in 1917, Oppenheimer and Rothschild presented their thesis on “electrocardiographic changes associated with myocardial involvement” at the annual meeting of the American Medical Association [2]. Extensive research in the following decades led to procession of modern cardiology. Still, therapeutic approaches to myocardial infarction remained for a long time without significant progress and patients were treated mainly with bed rest and opioids for decades. The first percutaneous transluminal coronary angioplasty constitutes a milestone in therapy of occluded coronary arteries and was introduced by Andreas Grüntzig in 1977 [3]. Many new technologies, from drug-eluting stents to interventional valve repair have been developed since.

Nowadays, “time” is still one of the biggest problems in modern care of myocardial infarction. Once irreversible cell death by ischemia has occurred, myocardial scarring leads to adverse remodeling, reduction in ventricular function, and serious adverse events, including arrhythmias, heart failure, and ultimately death. According to the 2015 Global Burden of Disease Study, cardiovascular diseases still represent the leading cause of death in noncommunicable diseases despite modern therapeutic approaches [4].

## 2. Stem Cells

Since the proliferating and self-healing capacity of cardiomyocytes in adults is limited, stem cell (SC) therapy has emerged as an attractive concept for heart repair and regeneration by restoration of cardiomyocytes and damaged myocardial tissue [5,6]. SCs are specified as undifferentiated cells possessing the ability to generate, sustain, and replace terminally differentiated cells via unlimited replication. They show two basic features, perpetual self-renewal and capability of differentiation into a specialized cell type under appropriate conditions [7,8]. SCs are commonly subdivided into two main entities, embryonic SCs (ESCs) and adult or somatic SCs. A third category of “embryonic-like” cells, the so-called induced pluripotent cells (iPSCs) that are genetically reprogrammed (by pluripotent transcription factors) has been added in the last years.

In cardiac regenerative medicine, the therapeutic use of pluripotent SCs (ESCs, iPSCs), possessing capacity to differentiate into all cell types of an organism including mesodermal derived cardiomyocytes, is limited mainly due to the risk of immune rejection, genetic instability, tumorigenic potential, low induction efficiency (iPSCs), and ethical issues (ESCs) [9,10,11]. The safety and efficacy of multipotent (differentiation into limited types of cells, e.g., mesenchymal SCs, cardiac SCs) or unipotent (differentiation into one cell type) adult SCs, however, have been intensively investigated for cardiac regenerative potential in clinical trials in the last 15 years. Many different types of adult SCs, distinguished by their origin and differentiation capacity, have been used, e.g., multipotent bone marrow derived SCs (BM-SCs) (including hematopoietic (HSCs), mesenchymal (BM-MSCs), endothelial stem cells), mesenchymal SCs (MSCs), skeletal myoblasts, and cardiac SCs (CSCs)) [12].

Skeletal myoblasts, myogenic progenitor cells residing beneath the basal lamina of myofibers, were the first cell type to be tested both in preclinical and clinical trials for cardiac regeneration. Lately, however, investigations diminished due to inconsistent therapeutic effect and observed risks of arrhythmias [13,14]. Likewise, multipotent CSCs, able to differentiate into cardiomyocytes, smooth muscle cells, and endothelial cells, have been used in clinical trials, but their limited quantity in adult hearts and difficult acquisition through invasive myocardial biopsies narrows their clinical-therapeutic use.

BM-derived cell types, especially the BM-MSCs, are attractive for therapeutic use in cardiac regenerative medicine due to their relatively easy accessibility and further isolation from autologous bone marrow or blood. Accordingly, their potential to optimize heart function, differentiation capacity into cardiomyocytes, angiogenic potential for a vascular regeneration, and favorable tolerance by the immune system holds additional benefit. However, exact mechanisms of cardiac repair by transplanted cells are still controversially discussed facing inconsistent results regarding therapeutic efficacy. Two main hypotheses exist: a) direct cardiomyogenic/vasculogenic differentiation, and b) indirect stimulation of the regenerative processes through paracrine, immunomodulatory effects by supply/secretion of soluble cytokines and growth factors [15,16].

## 3. History of Regenerative Therapies—Bone Marrow Cell Therapy

Research in the field of tissue engineering with cardiomyocytes has been conducted for more than 20 years. In 1993, Koh et al. already showed a significant long-term survival of cardiomyocyte grafts in adult murine hearts [17]. As early as 1998, Shin’oka and colleagues were able to produce “living” autologous grafts with vascular cells cultured on synthetic biodegradable tubular scaffolds and successfully implant them in pulmonary arteries of lambs [18]. In 2005 Shin’oka et al. published satisfactory follow-up data from human patients with corrected congenital heart disease, using tissue engineered vascular grafts derived from autologous bone marrow cells [19].

The landmark study by Orlic et al. in 2001 constituted a breakthrough. After coronary artery ligature, bone marrow cells of transgenic mice were injected in myocardium adjacent to infarcted areas. After nine days, in twelve out of thirty mice, newly formed tissue consisting of smooth muscle cells, endothelial cells, myocytes, and vascular structures was already able to replace an average of 68 ± 11% of the infarcted myocardium [20].

In 2002, the relatively small, but already in human subjects performed study TOPCARE-AMI (“Transplantation of Progenitor Cells and Regeneration Enhancement in Acute Myocardial Infarction”) individuals were randomized to receive either circulating blood-derived or bone marrow-derived progenitor cells directly into the culprit coronary artery 4.3 ± 1.5 days after acute myocardial infarction. Accordingly, significantly improved left ventricular function and markedly lower ventricular volumes were found in comparison to a nonrandomized matched control group. After four months, a significantly better coronary blood flow reserve, as well as significantly better viability obtained via FDG PET-CT was shown [21].

This was followed in 2004 by the BOOST-trial (“Intracoronary autologous bone-marrow cell transfer after myocardial infarction”) conducted by Wollert et al. The investigators compared sole optimal postinfarction medical therapy with intracoronary autologous bone marrow cell infusion 4.8 ± 1.3 days after acute myocardial infarction combined with optimal medical therapy. A marked improvement in left ventricular ejection fraction (LVEF; 6.7% vs. 0.7%) after a 6-month follow-up was demonstrated [22]. In 2006, a follow-up study of the BOOST-trial demonstrated that there was no significant difference in LVEF (obtained by cardiac MRI) after 18 months. However data suggested a more rapid increase in LVEF due to bone marrow cell infusion [23].

In their 2006 published, multicenter randomized placebo-controlled pilot-trial REPAIR-AMI (“Reinfusion of Enriched Progenitor Cells and Infarct Remodeling in Acute Myocardial Infarction”), Schächinger et al. showed significant improvement in myocardial performance by administration of bone marrow derived progenitor cells. Patients with acute ST-elevation myocardial infarction (STEMI) and successful percutaneous coronary intervention were allocated to bone marrow aspiration if significant LVEF-reduction (≤45%) was present. Of 204 patients, 101 individuals received intracoronary infusion of bone marrow cells (BMC) three to seven days after AMI. After four months, significant improvement in myocardial performance (LVEF obtained by “eye-balling”-method, improvement 5.5 ± 7.3% in the verum group vs. 3.0 ± 6.5% in placebo group) could be observed. Patients with LVEF ≤48.9% showed the greatest benefit. After one year significant reduction in clinical endpoints in the BMC group (death, relapse of myocardial infarction, renewed revascularization procedure) was shown [24].

Again in 2006, the ASTAMI trial (“Autologous Stem Cell Transplantation in Acute Myocardial Infarction”) was presented. A total of 50 out of 100 patients received autologous mononuclear bone marrow cells six days (median) after AMI into the affected coronary vessel. Patients in the control group received no bone marrow aspiration and no placebo-injections. Single-photon emission computed tomography (SPECT) and magnetic resonance imaging (MRI) were performed two to three weeks and six months after infarction. There was no significant difference in left ventricular function/-volume, infarct size or adverse events between groups [25].

In the same year, Janssens and colleagues showed no significant improvement in left ventricular function by BMC infusion in their double-blind, placebo-controlled study of 67 patients compared to the optimal medical treatment group. However, a slightly better LVEF and furthermore a significantly lower infarct size in the BMC group were demonstrated. Follow-up data were obtained using PET-CT and cardiac MRI. A benefit in terms of cardiac remodeling through BMC treatment was suspected by the authors [26].

A few years later, in 2015, the meta-analysis ACCRUE (“Meta-Analysis of Cell-based CaRdiac studies”) by Gyöngyösi et al. was published. A total of twelve randomized studies on intracoronary cell therapy for acute myocardial infarction were included. No effects of stem cell therapy on both MACCE (major adverse cardiac and cerebrovascular events) and secondary endpoints including death, re-infarction, and cerebrovascular stroke were reported. Furthermore, no effects of the therapy on LEVF or ventricular volumes were observed [27]. Therefore, it became evident that stem cells fail to engraft and survive in a reasonable extent in the injured adult myocardium under the conditions employed in clinical scenarios. While a number of different stem cell lineages, the influence of cell maturation through culturing, storage conditions, or application mode have been evaluated under in vitro and preclinical studies in vivo, the translational value has been lost on the way from bench to bedside.

The purpose of this review is to shed more light on methodological details and provide insights into processes at the extra- and intracellular levels. The importance of stem cell therapy after myocardial infarction has been discussed controversially in the past after distinct results in clinical application.

## 4. From Bed to Bench

**Figure 1:**
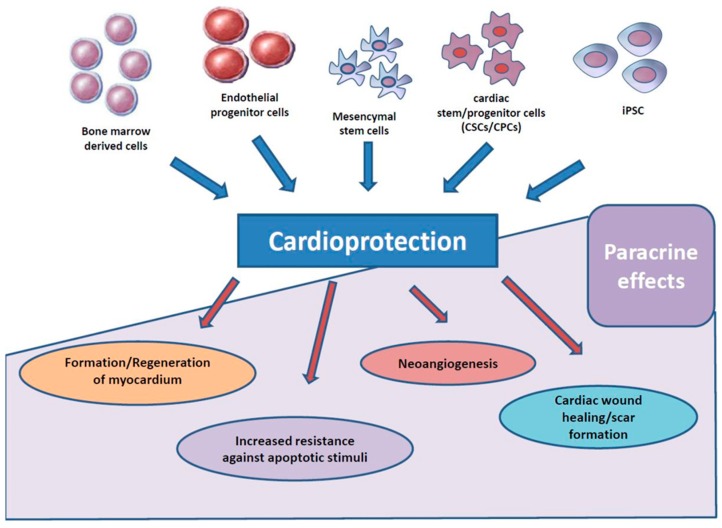
Comorbidities, timepoint of harvesting, cell separation methods, distinct media, additives, and distinct modes of delivery might contribute to distinct outcomes.

The distinct results in clinical stem cell trials initiated new research and the field of stem cell therapy went back from bed to bench. Comorbidities, timepoint of harvesting, cell separation methods, distinct media, additives, and distinct modes of delivery might contribute to distinct outcomes (Figure 1).

## 5. Methodological Issues

We and others postulated that, especially, differences in stem cell processing could contribute to contradictory outcomes: We evaluated specific differences in cell processing between ASTAMI and REPAIR-AMI [28]: Accordingly, REPAIR-AMI cells were stored at room temperature and coincubated with X-vivo medium and autologous serum, in the ASTAMI-trial cell storage was conducted at 4 °C, NaCl was used without any medium and instead of serum, heparinized plasma was added. Several issues arise with regards to addition of serum versus plasma, the use of X-vivo medium and distinct incubation temperatures used.

We could show that in vitro, incubation of stem cells at 4 °C (ASTAMI conditions) versus room temperature (REPAIR-AMI conditions) resulted in higher levels of IL-8 which is known to enhance cell survival and promote angiogenesis [28]. A further increase of incubation temperature to 37 °C led to even higher concentrations of IL-8 and might therefore help to optimize stem cell processing. The addition of autologous serum and X-vivo (REPAIR-AMI) led to higher IL-8 concentrations compared to plasma and NaCl (ASTAMI). Further, even coincubation of cell-free supernatant of REPAIR-AMI-like processed stem cells led to increased tube formation and enhanced migration of human endothelial cells (HUVECs), subsequently improving angiogenesis. These effects were at least partially mediated by phosphorylation/activation of the Akt/Erk pathway known to play a crucial role in acute cardioprotection [29].

## 6. The Paracrine Paradigm and Cardioprotection

**Figure 2:**
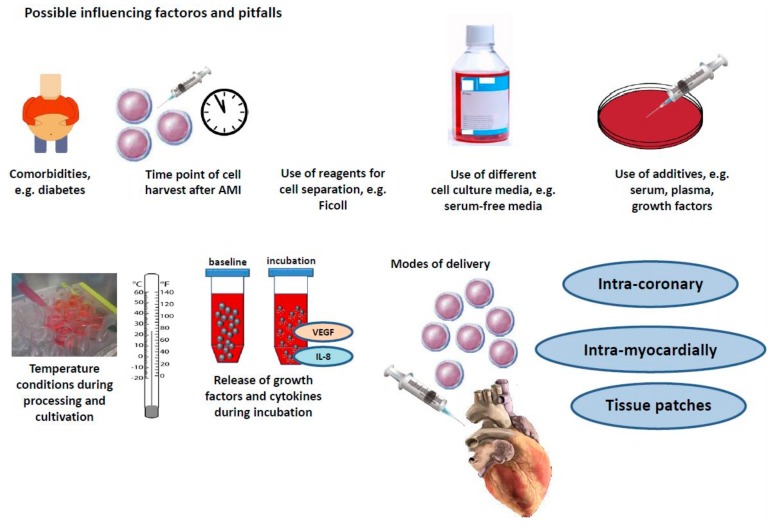
Several mechanisms for paracrine therapy after acute myocardial infarction (AMI) were proposed and evaluated.

This is of particular interest as in vitro data showed a reduction of hypoxia-induced rat cardiomyocyte cell death after addition of conditioned, cell-free, mesenchymal stem cell medium [30,31]. Therefore, a paracrine paradigm was proposed. Of note, cardioprotective and proangiogenic effects by solely adding supernatants of stem cells were demonstrated [30,31]. This concept of cell-free “stem cell therapy”, mitigating its effects through components of stem-cell supernatants gained support from other trials reporting surprisingly low rates of stem-cell incorporation after injection in human beings ranging from 90% to as low as 0%. This seriously called into question the whole very basic intellectual concept of stem cell therapy per se assuming injected stem cells to be incorporated and further differencing into cardiac cells in infarcted myocardial tissue [32,33,34]. Based on these doubts and new insights, several mechanisms for paracrine therapy after AMI were proposed and evaluated (Figure 2).

In 2005 “The Dying Stem Cell Hypothesis” was postulated by Thum et al., stating that stem cells used in therapeutic approaches are already apoptotic, initiating immunomodulatory mechanisms thereby inducing remodeling and potential cardioprotective effects [35,36,37]. In vitro, apoptotic peripheral blood mononuclear cells (APOSEC) after irradiation were shown to release significantly higher levels of IL-8 than untreated control cells [38,39]. Injection of APOSEC was further shown to increase LVEF and cardiac output in both a rodent and porcine model of AMI [40]. Again, these effects were regulated via the Akt/Erk pathway [40]. Therefore, a “danger” signal transferred by apoptotic cells might help to reduce infarct size by activating prosurvival and anti-apoptotic cascades and protect cells from hypoxia-induced cell death.

This is in line with other findings combining immunological and cardiovascular concepts. In patients suffering from acute myocardial infarction, evidence of decreased levels of dendritic cells in peripheral blood and an upregulation in inflammatory markers were shown [41]. Low levels of dendritic cells in infarcted myocardium are associated with a lower rate of fibrotic repair processes but increased numbers of mechanical complications, such as ventricular rupture [42]. These observations suggest recruitment of effector cells as a local immune response after AMI and in other pathologies, supporting their engagement in reparative mechanisms [43,44,45,46,47,48]. In a recent review, Frangogiannis pointed out the possible explanations of inflammatory processes following reperfusion on the cellular and molecular level. Dying cells initiate DAMPs (damage associated molecular patterns), leading to a cascade of inflammatory processes, as the activation of components of the cellular immune systems and chemokine release mainly from endothelial cells [49].

Despite overwhelming evidence for cell- and life-saving effects of reperfusion strategies, on a cellular level, “adverse effects” are observed: After restoring blood flow, reperfusion injury occurs and contributes to tissue damage [50]. Possible explanations range from oxidative stress and electrolyte imbalances to different metabolic and inflammatory pathways. In ischemia, an anaerobic state of cells leads to lower pH value, lactate accumulation, and intracellular sodium overload. Reperfusion restores cellular pH, leading to intracellular calcium-overload and exposure to reactive oxygen species (ROS), which subsequently initiates inflammatory processes [51]. Paracrine effects of stem cell therapy might be cardioprotective and helps to reduce reperfusion injury. Reperfusion injury and potential paracrine therapies are reviewed excellently elsewhere [52,53]. Given the distinct composition of stem cell supernatants following various cell processing protocols mediating specifically cardioprotective effects, this might very well partly explain the contradictory results in clinical trials [28].

## 7. Cytokines and Chemokines

In supernatants of stem cells, several proposed mediators of the described potential therapeutic effects of stem cell secretomes are present. Cytokines such as TNF-alpha, IL-6, or IL-8 were proposed as potential mediators of cardioprotection [54]: known proinflammatory cytokines were shown to increase resistance of cardiomyocytes to ischemia. Furthermore, IL-8 specifically is known to have effects on cell proliferation and angiogenesis [55].

Still, cytokines usually mediate very distinct effects and influence several pathways, giving them Janus-faced features which limits their therapeutic potential: High dose cytokine therapy in a “paracrine therapeutic” rationale in AMI is unfeasible as they come at high costs for physiologic systems also inducing inflammation and even cytokine storm in the worst case. Therefore, subtler mediators (with potentially less unfavorable side effects) of cardioprotection, angiogenesis, and healing after AMI came into the spotlight.

## 8. Exosomes

Exosomes are small, nanometer-sized vesicles released from various cell types into the bloodstream. Formerly, exosomes were considered to be cellular waste, but soon they were understood to play a crucial role in cell communication. Exosomes are proposed both as potential biomarkers and therapeutics in distinct pathologies [56]. Nearly all cells in the human body create and release small particles of 50–150 nm diameter, which are called exosomes. Exosomes are different from microvesicles with regards to both size (which is bigger in microvesicles, ranging from 100–1000 nm diameter) as well as production (exosomes are vesiculated within endosomal bodies, microvesicles are shed from the cell membrane).

The isolation and characterization of exosomes is still difficult, and distinct techniques such as chromatography, centrifugation, precipitation, and affinity-isolation are used [57,58]. Still, given their similar size and density, none of these techniques purifies exosomes completely from microvesicles, lipoproteins, and macromolecular complexes. Even the use of flow-cytometry is limited as exosomes are smaller than the light wavelength and although exosomes express surface proteins, they usually hint more towards the cell of origin than they are exosome-specific [57,59,60]. Exosome-free or serum-free cell culture medium was used to achieve higher purity [61].

Despite these methodological difficulties, exosomes have already been extensively evaluated and reviewed elsewhere in the field of cardiovascular medicine [57,62,63]. The injection of mesenchymal stem cell-derived exosomes was shown to reduce infarct size and increase cardiac ejection fraction in a mouse model of ischemia/reperfusion [64,65]. In a chronic ischemia rat model, Zhao et al. showed that, in rats, the intravenous injection of mesenchymal stem cell-derived exosomes led to increased cardiac function [66]. These effects might be mediated by angiogenic effects, but also by direct effects on the contractile capacity of myocardial cells [67,68]. These angiogenic effects were also seen in exosomes derived from hematopoietic stem cells [69]. Others even tried to tailor exosomes to be of greater cardiovascular advantage [70].

With regards to cardiac stem cells, exosomes were proposed to play an important role in the paracrine signaling and benefits seen in stem cell transplantation. Zhang et al. tried to precondition cardiac stem cells using exosomes [71]. The authors found that cardiac stem cells internalized exosomes. The use of exosomes increased tube formation, proliferation, and migration in cardiac stem cells. Further, in a rat model of myocardial infarction, preconditioned stem cells improved outcomes: survival and cardiac function were increased, whereas cardiac fibrosis was reduced [71].

## 9. Micro-RNAs: A New Player for an Old Concept?

Evaluating preconditioned cardiac stem cells using exosomes, the authors found preconditioning leading to a change in micro-RNA (miRNA) profiling [71]. Therefore, a significant part of the effects mediated by exosomes could be due to alterations in miRNAs. Recent evidence suggests that plasma exosomes also elicit cardioprotective effects in cardiac ischemia [72]. Notably, exosomes contain a broad spectrum of noncoding RNAs (ncRNAs), including micro-RNAs (miRNAs), which serve as intracellular signaling effectors and are considered major regulators of cell differentiation, metabolism, and development in all multicellular organisms [73,74].

MiRNAs are small (19–24 nucleotides), single stranded, endogenous, noncoding RNA sequences. By binding to certain messenger-RNAs (mRNAs), miRNAs prevent protein translation and thus regulate gene expression at a post-transcriptional level [75]. Some miRNAs are highly expressed in healthy cardiac tissue, and thus probably play a role in the maintenance of normal cardiac function (miR-1, miR-16, miR-27b, miR-30d, miR-126, miR-133, miR-143, and others) [76], whereas others have been associated with numerous disease entities, including coronary heart disease [75,77]. Interestingly, certain miRNAs are abundantly expressed in human embryonic stem cells (miR-302/367 cluster, miR-371, miR-372, and miR-373), where they play a role in the cell cycle and regulate pluripotency and cell differentiation [78]. The knowledge about these functions has put miRNAs in the focus of various study groups, which investigated their role and use in regenerative medicine, particularly in cellular reprogramming.

## 10. miRNAs in Cellular Reprogramming

**Figure 3:**
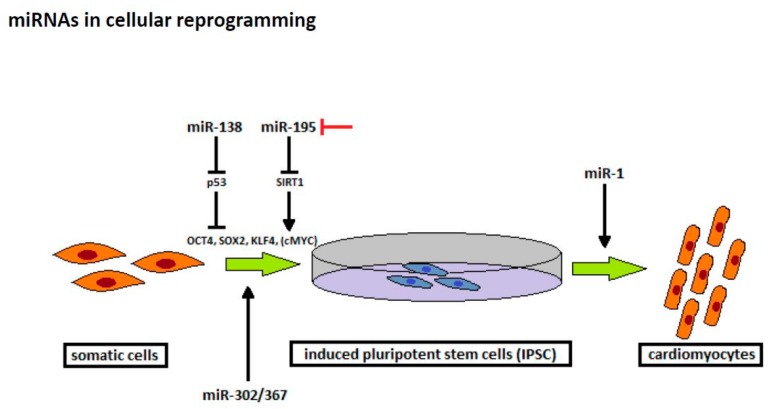
Novel strategies to improve the process of cellular reprogramming deferring from initial transcription factors.

In 2006, Takahashi et al. showed that differentiated somatic cells were reprogrammed into a pluripotent state by transduction of certain transcription factors, proving that cells can differentiate bilaterally [9]. The induced pluripotent stem cells (iPSC) show similar properties to embryonic stem cells (ESC), including the ability to differentiate into various cell lines, which offers a promising therapeutic approach in ischemic heart disease [79]. In their study, Takahashi et al. successfully generated iPSC from mouse fibroblasts, and later from human fibroblasts, by virus-mediated transduction of OCT3/4, SOX2, KLF4, and c-Myc (OSKM) [9,80]. C-Myc is an oncogene and OCT4, SOX2, and KLF4 are highly expressed in certain forms of cancer. Therefore, this method might carry substantial risk of tumorigenicity. Moreover, reprogramming efficacy using these factors is low but other studies investigated novel strategies to improve the process of cellular reprogramming deferring from initial transcription factors (Figure 3) [79].

Recent trials investigated the role of miRNAs in cellular reprogramming and found that some miRNAs promote generation of iPSC in combination with transcription factors [81,82,83]. A previous study reported that miR-138 improves the reprogramming efficiency by targeting the 3‘-untranslated regions of p53, a well-known tumor suppressor gene. In combination with OCT4, SOX2, and KLF4, miR-138 leads to a downregulation of p53 and its downstream genes, thereby improving iPSC generation [81]. In contrast, blockade of senescence-associated miR-195, which targets Sirtuin 1 (SIRT1) [82], and the deficiency in miR-34a, a target of p53 [83], can also significantly enhance reprogramming efficacy using the conventional reprogramming approach. Interestingly, a recent trial reported that cellular reprogramming can be conducted effectively and safely by using miRNAs. In their study, Anokye-Danso et al. found that the expression of the miR-302/367 cluster can reprogram mouse and human fibroblasts to iPSC without affecting exogenous transcription factors [84].

Besides in cellular reprogramming, miRNAs also seem to play a paramount role in the differentiation of pluripotent cells. In fact, a recent study by Lu et al. found, that miR-1 promotes the differentiation of iPSC to cardiomyocytes by modulating Wnt and fibroblast growth factor (FGF) pathways [85]. With the rapidly increasing knowledge on miRNA biology, the aforementioned findings may help to improve cellular reprogramming and allow an effective and safe method of iPSC generation in the future. Collectively, miRNAs may play a role in future regenerative cardiovascular therapy: 1) iPSCs are specifically be programmed and induced by a set of certain miRNAs internally in the adult heart or 2) miRNAs are used to improve incorporation and viability of externally transplanted stem cells.

## 11. Conclusions

Stem cell therapy is still an innovative and clinically needed therapeutic concept—although modern reperfusion therapy reduced myocardial infarction size—as myocardial cell death is still associated with both morbidity and mortality. Stem cell therapy was hyped in the early 2000s and as a consequence put into human application in a clinical setting very rapidly and most likely too early. The very distinct and contradictory results of clinical stem cell studies, such as ASTAMI and REPAIR-AMI, might at least partly be explained by differential cell processing of stem cells used in these clinical studies. In hindsight, it is astonishing why such basic and essential conceptual and methodological questions were not evaluated before clinical application. Tricks and tweaks on stem cell processing might improve stem cell incorporation and stem cell-mediated paracrine signaling, and hence outcomes.

With regards to paracrine signaling, there is a growing evidence that at least part of the therapeutic effects seen in clinical stem cell trials might be explained by these noncellular-mediated effects. The exact actors in this field remain unknown, but beside classical cytokines and chemokines, exosome-mediated cell signaling might play an essential role. 

Exosomes are difficult to investigate as their purification process is resource consuming and the resulting purity is not perfect. An essential part of exosomes constitutes miRNAs, which play both a role in paracrine cell signaling and stem cell signaling. 

Optimizing stem cell processing and a better understanding of paracrine signaling and its effect on cardioprotection and remodeling after AMI might both be promising research fields in the near future. In conclusion, in our opinion, the concept of stem cell therapy and its close relative, paracrine signaling, are not dead at all, but have just passed through birth pains.
